# Irc20 Regulates the Yeast Endogenous 2-μm Plasmid Levels by Controlling Flp1

**DOI:** 10.3389/fmolb.2020.00221

**Published:** 2020-11-19

**Authors:** Deena Jalal, Jisha Chalissery, Ahmed H. Hassan

**Affiliations:** Department of Biochemistry, College of Medicine and Health Sciences, United Arab Emirates University, Al-Ain, United Arab Emirates

**Keywords:** homologous recombination, Irc20, ubiquitin ligase, SUMO, Flp1, 2-μm plasmid

## Abstract

The endogenous yeast 2-μm plasmid while innocuous to the host, needs to be properly regulated to avoid a toxic increase in copy number. The plasmid copy number control system is under the control of the plasmid encoded recombinase, Flp1. In case of a drop in 2-μm plasmid levels due to rare plasmid mis-segregation events, the Flp1 recombinase together with the cell’s homologous recombination machinery, produce multiple copies of the 2-μm plasmid that are spooled during DNA replication. The 2-μm plasmid copy number is tightly regulated by controlled expression of Flp1 as well as its ubiquitin and SUMO modification. Here, we identify a novel regulator of the 2-μm plasmid, the ATPase, ubiquitin ligase, Irc20. Irc20 was initially identified as a homologous recombination regulator, and here we uncover a new role for Irc20 in maintaining the 2-μm plasmid copy number and segregation through regulating Flp1 protein levels in the cell.

## Introduction

The *Saccharomyces cerevisiae* 2-μm plasmid is an endogenous selfish circular plasmid that utilizes the cell’s own machinery to maintain copy number at 40–60 copies per haploid cell and segregation efficiency close to that of chromosomes (reviewed in Chan et al., 2013; Liu et al., 2014). The faithful segregation of the 2-μm plasmid to the daughter cells is coordinated by the plasmid partitioning system which comprises two plasmid encoded proteins, Rep1 and Rep2 and a *cis*-acting DNA element *STB* (Jayaram et al., 2004). Rep1 and Rep2 proteins form a complex at the *STB* locus, which acts in a manner similar to yeast centromeres where it recruits other host-encoded factors, such as Rsc2 and the cohesin complex (Wong et al., 2002; Yang et al., 2004), and helps in segregating the replicated plasmid into the mother and daughter cells. The copy number control system, on the other hand, ensures the copy number is maintained at 40–60 copies per cell. In rare mis-segregation events of plasmid molecules, the plasmid encoded recombinase Flp1 corrects the copy number by inducing a cut at the Flp recognition target (*FRT*s) initiating a double rolling circle replication mode which amplifies copy number with the help of the cellular homologous recombination (HR) machinery (Futcher and Cox, 1983; Futcher, 1986).

While innocuous at controlled levels, if the 2-μm plasmid reaches uncontrollable levels, it causes cell toxicities characterized by cold sensitivities and nibbled colony appearance (Chen et al., 2005; Burgess et al., 2007). The activity of Flp1 is tightly regulated at the level of gene expression through Rep1-Rep2 mediated inhibition (Murray et al., 1987), and by SUMO modification to regulate its function and mediate its ubiquitin dependent degradation, all preventing runaway increase in copy number (Xiong et al., 2009). Perturbations in several SUMO and ubiquitin pathway regulators cause increased copy numbers of the 2-μm plasmid, such as mutations in the deSUMOylating enzyme Ulp1 (Dobson et al., 2005), the SUMO conjugating enzyme Ubc9 (Burgess et al., 2007), the SUMO E3 ligases Siz1 and Siz2 (Chen et al., 2005), the SUMO targeted ubiquitin ligase (STUBL) complex Slx5-Slx8 (Burgess et al., 2007), as well as the ubiquitin conjugating enzyme Ubc4 (Sleep et al., 2001).

Irc20 is a recently identified HR regulator. It controls the spontaneous formation of recombination foci (Increased recombination centers) (Alvaro et al., 2007). It was further demonstrated that Irc20 promotes HR, and helps in directing it to the more error-free mode, synthesis dependent strand annealing (SDSA), as well as reducing cross-overs involving long-tracts of DNA (Miura et al., 2012). Irc20 harbors two conserved domains, a SNF2 ATP hydrolysis domain (Flaus et al., 2006), and a RING domain (Freemont, 2000; Richardson et al., 2013), which is characteristic for E3 ubiquitin ligases. Irc20 also contains at least two SIMs, allowing it to target SUMOylated proteins, including itself, hence considered a STUbL (Richardson et al., 2013).

In this study, we uncover a novel function for Irc20 in controlling the HR-dependent increase in 2-μm plasmid levels, and show that mutations in Irc20 affect the proper maintenance of the plasmid. Additionally, we show that Irc20 partly regulates the 2-μm plasmid stability and copy number by controlling the levels of the Flp1 recombinase.

## Materials and Methods

### Yeast Strains

The strains used in this study are listed in Supplementary Table 1. Gene tagging and deletions were done using single-step PCR mediated gene modifications (Longtine et al., 1998). Point mutations were introduced using *in vivo* site specific mutagenesis, *delitto perfetto* (Stuckey et al., 2011). Primers for gene modifications are listed in Supplementary Table 2. Strains lacking the 2-μm plasmid, designated [cir^0^], were derived from strains containing the 2-μm plasmid, [cir^+^], by expression of a defective Flp1 recombinase from the plasmid pBIS-GALkFLP-(URA3) (Addgene) (Tsalik and Gartenberg, 1998).

### 2-μm Plasmid Loss Assay

To monitor plasmid loss rates, [cir^0^] cells were transformed with pKan4 plasmid lacking *flp1* gene (a kind gift from Melanie Dobson), and selected on media containing the antibiotic Geneticin. Cells were then grown in liquid culture without antibiotic for 15 generations, followed by plating of equal amounts on YPD and YPD-Geneticin. Plasmid segregation efficiency was calculated as the ratio of number of colonies grown on geneticin plate (colonies harboring plasmid) to the number of colonies grown on YPD plate (total number of colonies) for *irc20Δ* and compared to WT strain.

2-μm harboring *His3MX6* inserted downstream of the *STB* locus were used to analyze the plasmid segregation efficiency for plasmids harboring the *FLP1* gene. Similar to pKan4, the plasmid segregation efficiency was calculated for Irc20 mutants and compared to WT strain.

### Analyzing 2-μm Plasmid Levels

Overnight cell cultures were pelleted and genomic DNA isolated using Wizard Genomic DNA purification kit (Promega). DNA was diluted to a concentration of 5 ng/μl and analyzed by quantitative real time PCR. Levels of 2-μm plasmid were measured using a sequence from the Y subtelomeric element as the reference. Primers for measuring 2-μm DNA and Y subtelomeric element are listed in the primer list. qPCR was carried out with QuantStudio 7 Flex (Applied Biosystems) using SYBR^TM^ green PCR master mix (Applied Biosystems). Each reaction contained 10 μl of SYBR Green Master Mix, 0.1 mM forward and reverse primers, 10 ng genomic DNA, and distilled H_2_O to a 20 μl final volume. PCR conditions were as follows: 1 cycle at 50°C for 2 min followed by 95°C for 10 min; and 40 cycles, each consisting of 95°C for 15 s, 60°C for 1 min. The fold change of the 2-μm number compared to that of wild-type yeast DNA was calculated by 2^–ΔΔCT^ methods. Five to nine independent colonies were analyzed for each strain, and qPCR done in duplicates or triplicates. Values were compared by the Student *t*-test.

### Extraction of Total Cellular Protein by TCA

Pellets from the yeast strains equivalent to an OD_600_ of 2.5–5 was collected by centrifugation, washed with 1 ml sterile water. Cells were then resuspended in cold (0.25 M NaOH/1% 2-mercaptoethanol) and incubated on ice for 10 min. 160 μl of 50% TCA was then added and incubated for another 10 min on ice. Precipitated proteins were centrifuged at 14,000 RPM for 10 min, washed with cold acetone and air-dried for 10 min. Proteins were resuspended in 100 μls 2X SDS-PAGE loading buffer, boiled for 5 min at 95°C, and centrifuged at 13,000 RPM for 5 min. 10–15 μls of supernatant were loaded on SDS-PAGE followed by western blotting.

### Monitoring Flp1 Levels

The *FLP1* gene was cloned into pRS416-Gal-RNQ1-YFP (Addgene; Douglas et al., 2008) using XhoI and BamH1 sites, to replace *RNQ1* gene. The resulting vector expresses C-terminal YFP-tagged Flp1 under *GAL1*-promoter, in which expression is induced when cells are grown in galactose, and is repressed when grown in glucose. This plasmid was transformed into [cir^0^] strains, and SC-Ura media was used for selection. Cells were grown overnight in SC-Ura broth containing raffinose, subcultured in the same media to get an exponentially growing culture. Cells were then switched to SC-Ura broth containing galactose and allowed to grow for 6 h to induce the expression of Flp1. Cells were then pelleted, resuspended in SC-Ura broth containing glucose, and allowed to grow to monitor the degradation of Flp1. Cells were collected at 45, 90, and 180 min, of total OD_600_ of 2.5, and whole cell extracts were prepared by TCA method. 10–15 μls of the whole cell extracts were loaded on 8% SDS-PAGE, and western blotting was done using anti-GFP antibody (Abcam). The blots were deprobed and then probed with Tubulin antibody (Abcam). The blots were scanned by Typhoon FLA 9500 and the bands were quantitated using ImageQuant TL software (GE Healthcare). The levels of Flp1 were calculated by comparing the band intensity at the time points indicated with the band intensity at 0 mins, normalized to the loading control.

### Southern Blotting for Analyzing 2-μm Species

15 μg of isolated DNA were ran on 1.2% agarose gel in 1X TBE containing 0.75 μg/ml chloroquine at 3 V/cm for 40 h at room temperature. The agarose gel was depurinated by incubating twice for 15 min in 0.25 M HCl, then denatured by incubating twice for 15 min in denaturation buffer (0.5 N NaOH, 1 M NaCl) and finally neutralized by incubating twice for 15 min in neutralization buffer (1 M Tris pH 7.5, 600 mM NaCl). DNA was then transferred to a positively charged nylon membrane overnight in 20X SSC (3 M NaCl, 0.3 Na citrate pH 7.0) by capillary transfer using a wick of Biorad filter paper (Brown, 1999). Membrane was crosslinked using UV, then hybridized with biotinylated probes designed to specifically detect the 2-μm plasmid in hybridization buffer (0.5 M Na_2_PO_4_ pH 7.2, 7% SDS, 1 mM EDTA) at 62°C overnight in hybridization oven. Membranes were washed twice for 15 min with 2X SSC, 0.1% SDS at 55°C followed by one wash with 1X SSC, 0.1% SDS at 55°C for 15 min. Detection of biotin labeled probe was done using Chemiluminescent Nucleic Acid Detection Module (Thermo Scientific^TM^) as per the instructions provided with the kit.

## Results

### Mutations in Irc20 Promote the Loss of the Highly Stable Endogenous 2-μm Plasmid

**FIGURE 1:**
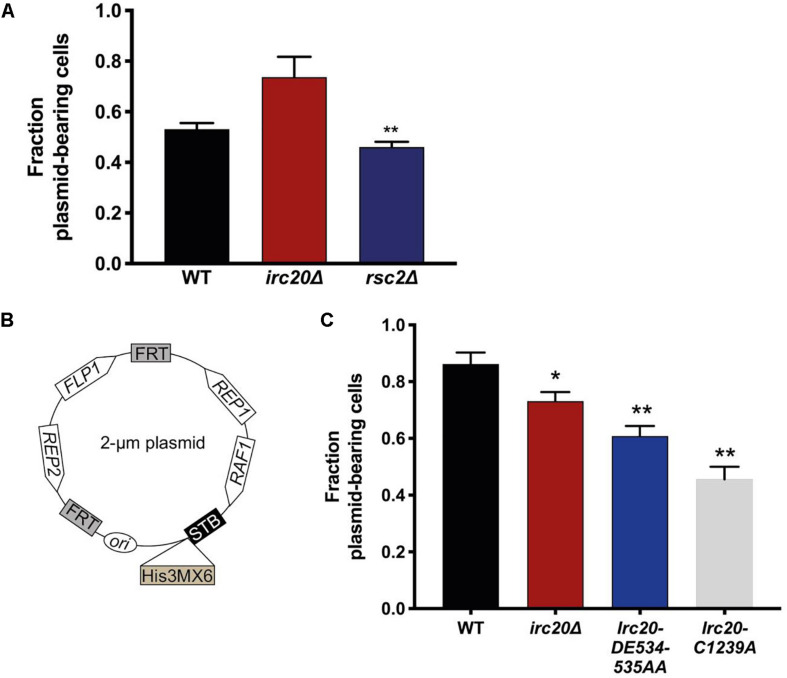
Mutations in Irc20 affect the segregation efficiency of the 2-μm plasmid. **(A)** The fraction of cells harboring pKan4 plasmid were calculated by counting the number of colonies on Geneticin plates compared to the number of colonies on non-selective plate (YPD) in three different independent cultures. **(B)** Schematic diagram of p2μm-His3MX6-STB, a modified form of the 2-μm plasmid where a *His3MX6* cassette is inserted downstream of the *STB* locus to allow for selection. **(C)** The fraction of cells harboring p2μm-His3MX6-STB plasmid were calculated by counting the number of colonies on SD-His plates compared to the number of colonies on non-selective plates (YPD) in three different independent cultures. Values are presented as mean ± SEM. One asterisk shows *p*-value < 0.05, two asterisks shows *p*-value < 0.01, as compared to WT.

We have frequently observed the generation of strains lacking the 2-μm plasmid when *irc20* was deleted (Supplementary Figure 1). In order to quantify the 2-μm plasmid segregation efficiency, we used pKan4, a modified form of the endogenous 2-μm plasmid, that is used for analyzing segregation efficiency, where it harbors a *KanMX4* selection marker and the partitioning system of the endogenous 2-μm plasmid, Rep1, Rep2, and the *STB*, but lacks the *flp1* gene (Pinder et al., 2013). This plasmid is designed this way because having a functional *FLP1* would obscure plasmid mis-segregation defects by correcting decreases in copy number (Sengupta et al., 2001; Pinder et al., 2013; McQuaid et al., 2019). To determine the rate of pKan4 loss, WT and *irc20*Δ [cir^0^] strains were transformed with pKan4, and transformants were initially grown in YPD medium containing the antibiotic Geneticin to select for plasmid retention. The proportion of plasmid-containing cells was then determined after 15 generations of growth in a medium that did not select for the retention of the plasmid (YPD), by comparing plating efficiency on solid YPD medium containing or lacking Geneticin. We used *rsc2*Δ mutant as a positive control for this experiment as it is known to have lower plasmid retention rates because of the role of Rsc2 in the 2-μm plasmid partitioning (Wong et al., 2002; Pinder et al., 2013). To our surprise, the segregation efficiency of the pKan4 plasmid was not reduced in the *irc20*Δ strain (Figure 1A). The *rsc2*Δ mutant, however, showed less pKan4 plasmid segregation efficiency, at a similar level to a previous report (Pinder et al., 2013).

To reconcile the discrepancies in the 2-μm plasmid segregation efficiency, we constructed a new 2-μm plasmid harboring all the plasmid encoded genes, including *FLP1*, and a *His3MX6* cassette (which allows the cells to grow on media lacking Histidine) inserted downstream of the *STB* locus (Figure 1B). To determine the rate of p2μm-His3MX6-STB loss, WT and *irc20*Δ [cir^0^] strains were transformed with the plasmid and were initially grown in SD-His medium to select for the plasmid retention. The proportion of plasmid-containing cells was determined as discussed earlier. Using this construct, we were able to detect lower plasmid segregation efficiency in the *irc20Δ* mutant strain as compared to WT (Figure 1C). To identify the functional domain in Irc20 responsible for plasmid loss, we introduced point mutations in the ATPase domain and in the RING finger domain, *Irc20-DE534-535AA* and *Irc20-C1239A*, respectively, that abolishes either activity (Richardson et al., 2013). These point mutants were transformed with the p2μm-His3MX6-STB and tested for plasmid retention, and similar to *irc20*Δ showed lower plasmid segregation efficiency than WT. The 2-μm plasmid segregation efficiency was even lower when cells harbor point mutations in either the ATPase or the ubiquitin ligase domain of Irc20, than in the *irc20*Δ mutant. The maximum reduction in plasmid segregation efficiency was seen in the ubiquitin ligase mutant (*Irc20-C1239A*), where the fraction of cells bearing the plasmid reached 0.5 as compared to WT where it was 0.85. It is worth-noting that the pKan4 and the p2μm-His3MX6-STB differ in the plasmid background, where pKan4 contains bacterial maintenance sequences and p2μm-His3MX6-STB does not. This accounts for the difference in plasmid segregation efficiencies between both in WT strain, as introduction of DNA sequences in the endogenous 2-μm plasmid is known to affect its segregation efficiency (Wong et al., 2002; Pinder et al., 2013).

### Irc20 Controls Copy Number Amplification of the 2-μm Plasmid by Regulating the Flp1 Protein Levels in the Cell

**FIGURE 2:**
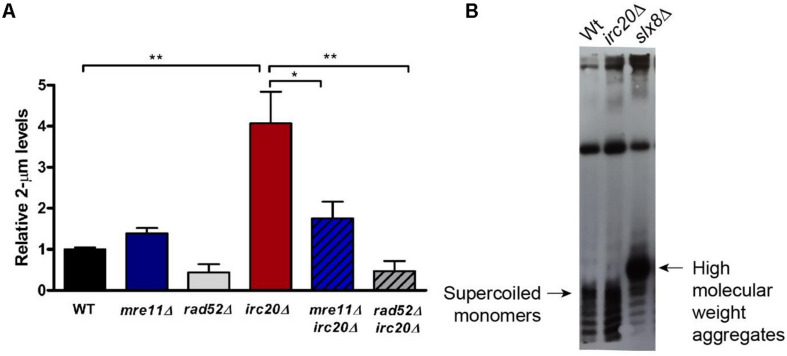
The absence of Irc20 increases the endogenous 2-μm plasmid levels without forming HMW aggregates. **(A)** The levels of endogenous 2-μm plasmids in WT, *irc20Δ, mre11Δ, rad52Δ, mre11Δirc20Δ*, and *rad52Δirc20Δ* mutants were measured by real-time PCR using primers specific to the 2-μm plasmid DNA sequence and quantified relative to Y-subtelomeric regions. *irc20Δ* mutant showed 4-fold higher levels of the 2-μm plasmid compared to WT. Double mutants of *irc20* with HR repair factors, *mre11* and *rad52*, showed significantly reduced 2-μm plasmid levels compared to single *irc20Δ* mutant. Five to nine independent cultures were analyzed and values are presented as mean ± SEM. Statistical significance was calculated by two-tailed Student *t*-test for unpaired samples. One asterisk shows *p*-value < 0.05 and two asterisks show *p*-value < 0.01, as compared to WT or *irc20Δ* as indicated. **(B)** Southern blot analysis of DNA isolated from WT, *irc20Δ* and *slx8Δ* using biotinylated probes specific to 2-μm sequence. The hybridized probes were then visualized using Chemiluminescent Nucleic Acid Detection Module (Thermo Scientific^TM^).

The elevation of the 2-μm plasmid in several SUMO pathway mutants, such as *siz1*Δ*siz2*Δ double mutant, and STUbLs, such as *slx5*Δ and *slx8*Δ, have already been reported and is considered to be responsible for the nibbled colony appearance and cold sensitivities observed in these mutants (Chen et al., 2005). Even though *irc20*Δ does not show any growth sensitivities, there seems to be a link between Irc20 and the plasmid amplification system, specifically Flp1, underlying the defective segregation efficiency of the 2-μm plasmid observed in *irc20* mutants. To understand this further, we measured the levels of the endogenous 2-μm plasmid by isolating DNA from WT and *irc20*Δ strains, and quantified by real time PCR using 2-μm specific primers normalized to a genomic control region, Y-subtelomeric regions. Our results show 3 to 4-fold higher 2-μm plasmid levels in *irc20*Δ mutant (Figure 2A). We next measured whether the elevation of 2-μm plasmid levels in *irc20*Δ was dependent on the HR pathway, since the Flp1 mediated copy number amplification of the 2-μm plasmid depends on the cellular HR machinery (Futcher, 1986; Xiong et al., 2009). This was done by analyzing the 2-μm plasmid levels in *irc20*Δ*rad52*Δ and in the *irc20*Δ*mre11*Δ double mutants. We observed a significant reduction in the endogenous 2-μm levels when loss of the *irc20* gene was coupled with deletions in *mre11* and *rad52* HR factors. The reduction was more pronounced in double mutants with *rad52*, possibly reflecting some residual HR events occurring in the absence of Mre11. These results indicate that the amplification of the 2-μm copy number in *irc20*Δ mutant is due to improperly regulated HR events.

We next tested whether the copy number amplification in *irc20*Δ is associated with the formation of high molecular weight aggregates (HMW) as seen in several SUMO pathway mutants (Xiong et al., 2009). The 2-μm plasmid forms isolated from WT and *irc20*Δ mutant were analyzed by Southern blotting using biotinylated probes specific to the 2-μm sequence. Despite the increased copy number observed in *irc20*Δ mutant, we did not observe aberrant species of the 2-μm plasmid like those observed in the *slx8*Δ mutant (Figure 2B). This is, however, consistent with the lack of growth defects and cold sensitivities in the *irc20*Δ mutant.

**FIGURE 3:**
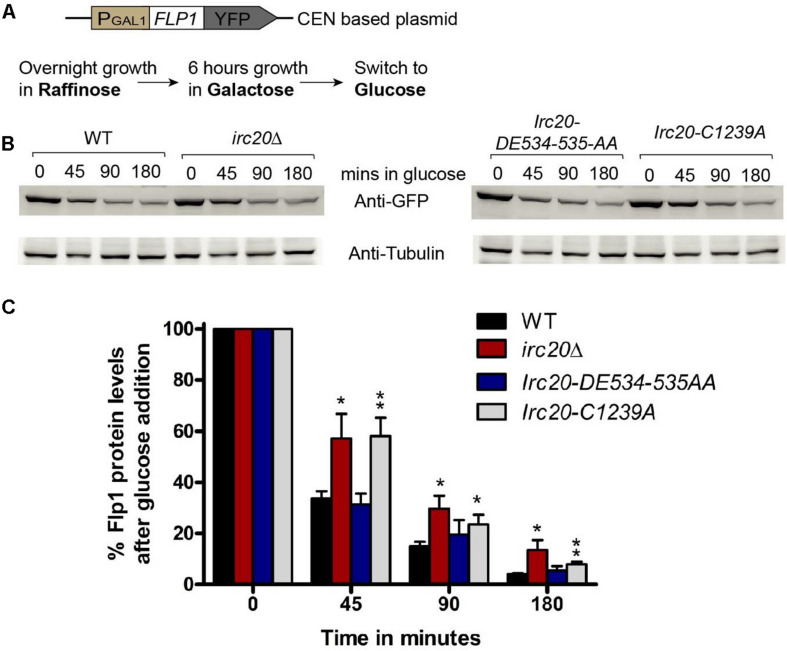
Loss of Irc20 ubiquitin ligase activity increases the levels of Flp1. **(A)** WT, *irc20Δ, Irc20-DE534-535AA* and *Irc20-C1239A* [cir^0^] mutants transformed with a centromeric plasmid (pRS416) expressing YFP-tagged Flp1 under *GAL1*-promoter were grown for 6 h in media containing 2% galactose to induce expression of Flp1 and then transferred to media containing 2% glucose to monitor the degradation of Flp1. **(B)** Whole cell extracts of cells grown for 45, 90, and 180 min in media containing glucose were analyzed for Flp1 levels by western blotting using anti-GFP antibody. **(C)** Band intensities were normalized to Tubulin band signals, and quantified as a percentage of signals before glucose addition. Values are the average of at least four replicate experiments, and are presented as mean ± SEM. Statistical significance was calculated by two-tailed Student *t*-test for unpaired samples. One asterisk shows *p*-value < 0.05 and two asterisks show *p*-value < 0.01, as compared to WT.

Since HR only participates in copy number amplification initiated by Flp1 incision at the *FRT*s, we tested whether there could be an increased Flp1 activity in the absence of Irc20. To test this, the *FLP1* gene was cloned into a centromeric plasmid (pRS416), under the *GAL1*-promoter with a YFP-tag at the C-terminus (Figure 3A). The repressible *GAL1*-promoter allows for shutting off expression, to monitor the existing levels of a protein without interference from newly expressed ones. This plasmid was transformed into WT, *irc20*Δ, *Irc20-DE534-535AA* and *Irc20-C1239A* [cir^0^] strains and were grown overnight in raffinose containing media. To induce Flp1 expression, the cells were grown for 6 h in galactose, then transferred to glucose-containing medium, to switch off expression and monitor the degradation of Flp1 in the presence or absence of Irc20. We observed less degradation of Flp1 in cells lacking Irc20 (Figures 3B,C). The levels of Flp1 reduced to 31–34% by 45 min in wild-type and Irc20-DE534-535AA mutant following switching to glucose media, whereas it remained at 57–58% levels in the *irc20*Δ and *Irc20-C1239A* mutants. This suggests that the increased copy number of the 2-μm plasmid in *irc20*Δ mutant could be, at least partly, due to increased Flp1 levels. We next measured the steady state Flp1 levels in WT and *irc20*Δ using a Myc tagged Flp1 to understand how the degradation rate reflects on the steady state level of Flp1 when expressed from its native promoter. Similar to a previous report (Chen et al., 2005), we found that even though C-terminal tagging of Flp1 does not affect its activity *in vitro* (Chen and Rice, 2003), the Flp1 tagging reduces the accumulation of 2-μm plasmid in *irc20*Δ (1.5-fold) compared to unmodified 2-μm plasmid (4-fold) (Supplementary Figure 2A). However, even though 2-μm plasmid levels were elevated in *irc20*Δ, we found similar Flp1 levels in both WT and *irc20*Δ when whole cell extracts were analyzed (Supplementary Figure 2B). This is likely because the expression of Flp1 from its native promoter is rapidly repressed when the 2-μm plasmid levels rise. Altogether, Irc20 seems to play a role in facilitating the degradation of Flp1 in the cell, and acts as an additional control over Flp1 to prevent the toxic increase in 2-μm plasmid copy number.

## Discussion

Irc20 was identified in a screen for genes causing increased recombination centers when deleted (Alvaro et al., 2007). The absence of Irc20 was shown to cause a general reduction in SDSA and crossover recombination, suggesting a regulatory role for Irc20 in recombination (Miura et al., 2012). Irc20 has two conserved domains, a SNF2 ATP hydrolyzing domain and a RING domain characteristic for E3 ubiquitin ligases, in addition to at least two SIMs, suggesting a role as a STUbL. E3 ubiquitin ligases including STUbLs play a regulatory role on various proteins in diverse cellular pathways.

Here we expand the cellular role of Irc20 to include the proper maintenance of the endogenous 2-μm plasmid. First, we show that mutations in *irc20* frequently caused the loss of the otherwise highly stable 2-μm plasmid, in a manner dependent on Flp1 expression, and not through the control of the Rep1-Rep2-*STB* pathway. In addition to a role in plasmid segregation, we show that despite the lack of cell sensitivities associated with increased 2-μm plasmid levels, *irc20*Δ mutant increased 2-μm plasmid levels 4-folds compared to the WT. Since Flp1 overexpression causes an amplification of 2-μm plasmid copy number (Reynolds et al., 1987; Xiong et al., 2009), we checked the role of Irc20 in regulating Flp1 protein levels. We monitored the YFP-tagged Flp1 levels in WT and *irc20*Δ after expressing from a repressible promoter and our results showed higher levels of Flp1 in *irc20*Δ, even though the steady state levels of the protein is not affected when expressed from its native promoter. This could be because of the autoregulatory nature of 2-μm plasmid genes, where the expression of Flp1 is rapidly repressed when the 2-μm plasmid levels rise, due to an increase in Rep1-Rep2 complex that act as repressor at the Flp1 promoter (Murray et al., 1987; Reynolds et al., 1987; Som et al., 1988). Altogether, our data suggests that Irc20 fine-tunes Flp1 levels, thereby controlling the amplification of the 2-μm plasmid copy number.

The reduced turnover of Flp1 when *irc20* is mutated is similar to that observed in *siz1*Δ*siz2*Δ, *slx5*Δ and *slx8*Δ which affect the SUMOylation and/or the subsequent ubiquitin dependent degradation of Flp1 (Ma et al., 2019). The resulting increase in copy number and formation of HMW aggregates is, however, different in case of *irc20* mutants. The increase in 2-μm plasmid copy number observed in *irc20*Δ mutant is not as high as that in other SUMO pathway mutants (10 to 30-folds) (Xiong et al., 2009), and the increased Flp1 induced recombination does not lead to the formation of HMW aggregates. We thus speculate that improper resolution of daughter plasmids during the Flp1-dependant amplification of copy number is not solely due to increased Flp1 levels or its deficient SUMOylation, but due to an additional role for Siz1, Siz2, Slx5, and Slx8 in regulation of HR. Several HR proteins are known to be SUMOylated during HR repair, both facilitating the assembly of repair foci, and inducing a Slx5-Slx8 control on HR protein levels (Psakhye and Jentsch, 2012). The increase in Flp1-induced nicks concomitant with a lack of regulatory control over HR, would lead to the aberrant processing of the replicated plasmids into monomers in daughter cells, thus HMW aggregate formation. This is consistent with that *slx5Δ* and *slx8Δ* still produces toxic 2-μm plasmid forms even when Flp-SUMO is expressed, despite being attenuated in strand cleavage (Ma et al., 2019). The reason would be deficient regulation on HR proteins. In the case of *irc20* mutants, however, the increase in Flp1 protein levels is associated with a reduction in HR, thus would only cause a small increase in 2-μm plasmid levels. A similar modest increase in copy number without significant growth sensitivities was previously reported for the ubiquitin conjugating enzyme Ubc4 (Sleep et al., 2001).

The Flp1-dependent increase in 2-μm copy number in *irc20*Δ does not explain, and somewhat contradicts the low 2-μm plasmid segregation efficiency observed in the *irc20*Δ mutant. We can only speculate that the imprecise repair in the absence of Irc20 (Miura et al., 2012), causes mutations in the regions surrounding the *FRT*s possibly the *STB*, thus affecting the integrity of the partitioning system, in a manner dependent on Flp1-induced incision, and HR repair. Altogether, we believe that Irc20 regulates the copy number of the 2-μm plasmid through regulating Flp1 levels, possibly by assisting its degradation. Irc20 also seems to be important in the precise recombination, and its absence could lead to mutations affecting the plasmid’s segregation efficiency.

## Data Availability Statement

All datasets generated for this study are included in the article/Supplementary Material.

## Author Contributions

AH obtained the funding and conceived and supervised the research. DJ designed and performed the experiments and analyzed the data with help from JC throughout. Experiments in Figures 2B, 3 and Supplementary Figure 2 were performed by JC as well as plasmid cloning and some strain constructions. DJ wrote the manuscript with contribution from JC and AH. All authors contributed to the article and approved the submitted version.

## Conflict of Interest

The authors declare that the research was conducted in the absence of any commercial or financial relationships that could be construed as a potential conflict of interest.
