# Supplementary material for: Irc20 Regulates the Yeast Endogenous 2-μm Plasmid Levels by Controlling Flp1
Source: Front Mol Biosci. 2020 Nov 19;7:221. doi: 10.3389/fmolb.2020.00221 (PMC7710549; doi:10.3389/fmolb.2020.00221)
Supplement: Supplementary file 1 [file Data_Sheet_1.docx]

# Supplementary data

## Supplementary Tables

### Supplementary Table 1: Yeast strains used.

| **Strain** | **Description** | **Source** |
| --- | --- | --- |
| BY4741 (WT) | MATa; *his3*Δ*1*; *leu2*Δ*0*; *met15*Δ*0*; *ura3*Δ*0* | Euroscarf |
| ADJ9 | BY4741; *irc20*Δ * | This study |
| ADJ40 | BY4741; *irc20DE534-535AA* * | This study |
| ADJ107 | BY4741; *irc20C1239A* * | This study |
| ADJ77 | BY4741; *rsc2*Δ::His3MX6 | This study |
| ADJ78 | BY4741 [cir^0^] | This study |
| ADJ79 | ADJ9 [cir^0^] | This study |
| ADJ82 | ADJ40 [cir^0^] | This study |
| ADJ83 | ADJ107 [cir^0^] | This study |
| ADJ80 | BY4741; *irc20*Δ::His3MX6 | This study |
| AJC22 | BY4741; *mre11*Δ::KanMX6 | This study |
| Y00540 | BY4741; *rad52*Δ::KanMX6 | Euroscarf |
| ADJ105 | AJC22; *irc20*Δ::His3MX6 | This study |
| ADJ106 | BY4741; *rad52*Δ::KanMX6 *irc20*Δ::His3MX6 | This study |
| ADJ99 | BY4741; *slx8*Δ::KanMX6 | This study |

*: delitto perfetto

### Supplementary Table 2: Primers used in this study

| **Name** | **Sequence** |
| --- | --- |
| DJ8 Deletion FP | AGGAATGAACTCCAGGAAAGGCCAACTTATTCGACGGTAATATATTAAAATATTTAATCTTTTGAATTTTTATATAAACG |
| DJ9 Deletion RP | CGTTTATATAAAAATTCAAAAGATTAAATATTTTAATATATTACCGTCGAATAAGTTGGCCTTTCCTGGAGTTCATTCCT |
| DJ34 CoRE DE534-535AA FP | ATGACTATTCTTCACCGTTAGCTTTGATGCAGTTTTATAGAATCATTCTGTTCGTACGCTGCAGGTCGAC |
| DJ35 CoRE DE534-535AA RP | AAACTCGTGCATTTTGCAGAATATGTTGATGAACTACGTAGCATTTGAACTAGGGATAACAGGGTAATCCGCGCGTTGGCCGATTCAT |
| DJ36 DE534-535AA FP | ACCGTTAGCTTTGATGCAGTTTTATAGAATCATTCTGGCTGCTGTTCAAATGCTACGTAGTTCATCAACATATTCTGCAA |
| DJ37 DE534-535AA RP | TTGCAGAATATGTTGATGAACTACGTAGCATTTGAACAGCAGCCAGAATGATTCTATAAAACTGCATCAAAGCTAACGGT |
| DJ42 CoRE C1239A FP | TGTCCACACTTAATGATAGCACCTATTTCAACTTCTCCCAAACAGATAGATTCGTACGCTGCAGGTCGAC |
| DJ43 CoRE C1239A RP | AATTTGTCCAGATTGAAAGACACATTAAACGATAATCAAATATTGAGCTAGGGATAACAGGGTAATCCGCGCGTTGGCCGATTCAT |
| DJ46 C1239A Foligo | GATTGAAAGACACATTAAACGATAATCAAATATTGAGCGCTTCTATCTGTTTGGGAGAAGTTGAAATAGGTGCTATCATT |
| DJ47 C1239A Roligo | AATGATAGCACCTATTTCAACTTCTCCCAAACAGATAGAAGCGCTCAATATTTGATTATCGTTTAATGTGTCTTTCAATC |
| JC30 Del Rad52 FP | CAAGAACTGCTGAAGGTTCTGGTGGCTTTGGTGTGTTGTTG CGGATCCCCGGGTTAATTAA |
| JC31 Del Rad52 RP | TAA TGA TGC AAA TTT TTT ATT TGT TTC GGC CAG GAA GCGTTGAATTCGAGCTCGTTTAAAC |
| JC54 Del Mre11 FP | GACGCAAGTTGTACCTGCTCAGATCCGATAAAACTCGACTCGGATCCCCGGGTTAATTAA |
| JC55 Del Mre11 RP | GTT ATA AAT AGG ATA TAA TAT AAT ATA GGG ATC AAG TACAAGAATTCGAGCTCGTTTAAAC |
| DJ78 Y sub-telomeric element FP | ACAATGGCCTTCGACTCTGGTTC |
| DJ79 Y sub-telomeric element RP | ATCACAGCCCGAAGAAGCACT |
| DJ80 2-µm qPCR FP | CACAAGATAGTACCGCAAAACGA |
| DJ81 2-µm qPCR RP | CACCTTTGCTGCTTTTCCTTAATT |
| DJ82 2-µm presence PCR FP | ACAGCGCTGATATACAATG |
| DJ83 2-µm presence PCR RP | CTGTCGGCTATTATCTCCG |
| DJ91 rsc2 deletion FP | AGAACCAGACGAAGCGGAGAATATTCTACATTGACAGTGCCGGATCCCCGGGTTAATTAA |
| DJ92 rsc2 deletion RP | GGAAGATATTATGCTGCCATTGCTTTTACAATAAAGGTGAGAATTCGAGCTCGTTTAAAC |
| DJ112 del-myc-FLP1 RP | gtacgcatttaagcataaacacgcactatgccgttcttctGAATTCGAGCTCGTTTAAAC |
| DJ114 myc FLP1 FP | GGTACTAGACTACCTTTCATCCTACATAAATAGACGCATACGGATCCCCGGGTTAATTAA |
| DJ117 FLP1 Xba1 FP | ACGATCTCTAGAAAAAAAATGTCTATGCCACAATTTGGTATATT |
| DJ118 Flp1 BamH1 RP | ACGATCGGATCC TATGCGTCTATTTATGTAGG |
| DJ124 His3MX6 STB | AGGCATCCCCGATTATATTCTATACCGATGTGGATTGCGCCGGATCCCCGGGTTAATTAA |
| DJ125 His3MX6 STB | GAAGAATCATCAACGCTATCACTTTCTGTTCACAAAGTATGAATTCGAGCTCGTTTAAAC |
| DJ126 Southern 2um probe 1 | Biotin-GCCGTGGCCAGGACAACGTATACTCATCAGA TAACAGCAATACCTGATCACTACTTCGCACTAGTTTCTCGGTACTATGC |
| DJ127 Southern 2um probe 2 | Biotin-ATATGATCCAATATCAAAGGAAATGATAGCAT TGAAGGATGAGACTAATCCAATTGAGGAGTGGCAGCATATAGAACAGC |

## Supplementary Method

## Measuring the presence of the endogenous 2-µm plasmid

Cells harboring the 2-µm plasmid were grown over night in YPD, then plated on YPD at a dilution that allows for single colonies to be picked. 8 colonies from each strain were analyzed by colony PCR using primers specific for 2-µm plasmid. Colonies were resuspended in 30 µl lyticase (50 U/ml) in 0.2 ml PCR tubes, and incubated at 37°C for 30 minutes, followed by 95°C for 10 minutes. 2 µl of the lysed cells were analyzed by PCR using Taq polymerase in a 20 µl reaction. Samples were loaded on 1% Agarose gel and visualized by EtBr.

## Supplementary Figures

###
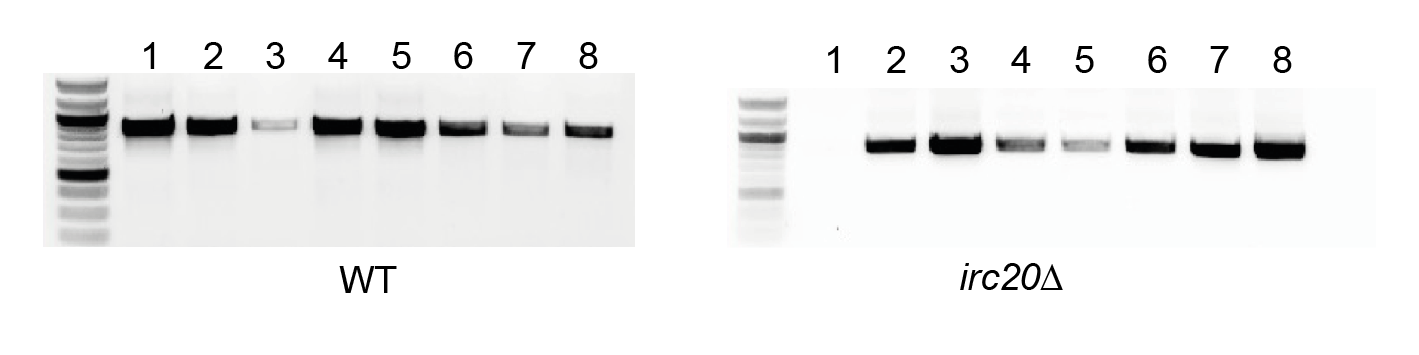
Supplementary Figure 1: Deletion of *irc20* affects the segregation efficiency of the endogenous 2-μm plasmid.

Eight single colonies from WT and *irc20*Δ were analyzed by colony PCR to detect the presence of the endogenous 2-µm plasmid after overnight growth. Occasional loss of the endogenous 2-µm plasmid was observed in *irc20*Δ mutant (1 in 8 colonies).


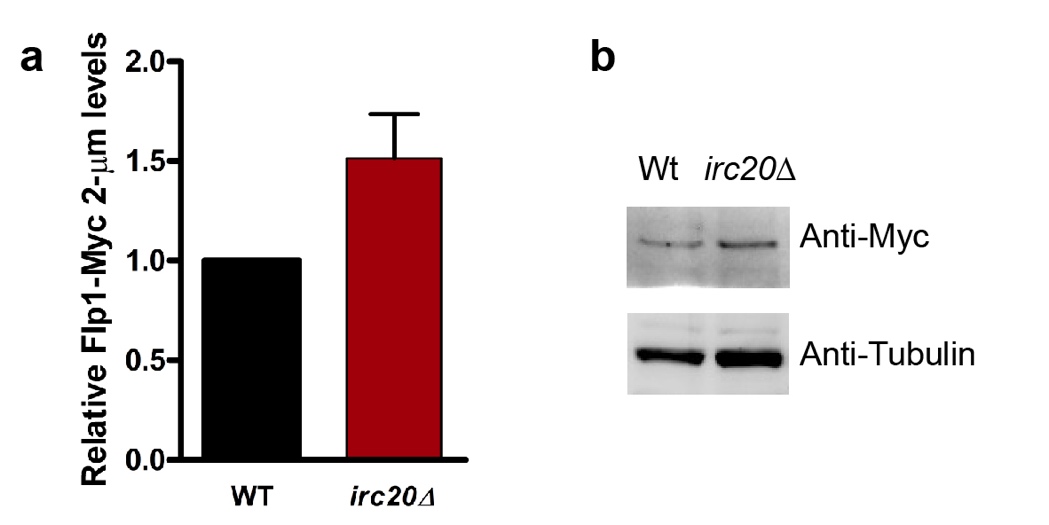


### Supplementary Figure 2: Flp1-Myc protein levels is similar in WT and *irc20*Δ.

(a) The levels of p2µm-Flp1-13Myc:His3MX6 plasmids in WT and *irc20*∆ were measured by real-time PCR using primers specific to the 2-µm plasmid DNA sequence and quantified relative to Y-subtelomeric regions. Three independent cultures were analyzed and values are presented as mean ± SEM. *irc20∆* mutant showed 1.5-fold higher levels of the Flp1-Myc tagged 2-µm plasmid compared to WT. (b) Whole cell extracts were analysed by western blotting using anti-Myc antibody and anti-tubulin antibody. Flp1 levels are similar in WT and *irc20*Δ*.*
